# Krauzlis' Strange Inversion of Reasoning

**DOI:** 10.3389/fnsys.2018.00034

**Published:** 2018-07-20

**Authors:** Domenico Guarino

**Affiliations:** Centre National de la Recherche Scientifique, Unité de Neurosciences Information et Complexité, Paris, France

**Keywords:** attention, multiple-draft model, homunculus, cell assembly, basal ganglia, superior colliculus

“Attention” is a central faculty of the mind (Baars, 1988; Minsky, 1988). It has been early proposed that it is a process selecting some of the available information to focus on enhanced processing (Deutsch and Deutsch, 1963; Treisman and Gelade, 1980). Contrasting this approach, Richard J. Krauzlis recently (2014; 2017) proposed that attention is an *effect* of selection processes instead of being their *cause*. In this article, I will argue that Krauzlis' view realizes what Dennett (2009) calls a “strange inversion of reasoning,” in which the competence of selecting behaviors does not need the comprehension of any attentive homunculus. In order to make the equivalence, I will detail an interface mapping between a philosophical position (Dennett's Multiple-Draft model, 1991) and a neuroscientific position (Krauzlis' attention as an effect). Such a mapping is meant to encourage a scientifically grounded dialogue between the two disciplines.

According to the original feature-integration theory of visual attention, “features are registered early, automatically, and in parallel across the visual field, while objects are identified separately and only at a later stage, which requires focused attention” (Treisman and Gelade, 1980, p. 98). The selection mechanism of attention, and its corresponding neural correlates, have been henceforth the target of further research. Crick (1984) proposed, with his “searchlight” hypothesis, that the thalamic reticular nucleus controls the focus of attention, whose content is represented at the cortical level. In fact, reticular cells receive connections from both feedforward thalamic and feedback cortical pathways, and they also inhibit each other, thus having the necessary circuitry to implement competition among their afferents (Sherman and Guillery, 2001). Over time, several other brain areas have been implicated in attentive behavior (Krauzlis et al., 2013): sensory and parietal cortex, frontal cortices like the frontal eye field, with a growing role for the superior colliculus (SC). Recently, Lovejoy and Krauzlis (2010) locally and reversibly suppressed the SC of monkeys performing a visual detection task. For stimuli placed in the affected part of SC, monkeys' performance was drastically reduced. Using the searchlight hypothesis, the conclusion would be that SC selects which part of the visual field has to be the target of attention, by supplying the ascending pathway to cortex with a saliency map (Koch and Ullman, 1987). Thus, the next step (Zénon and Krauzlis, 2012) consisted in transiently inactivating the SC during a detection task and measuring responses in corresponding visual cortical areas, with the prediction that cortical activity would be reduced due to SC inactivation. Surprisingly, the inactivation of SC did impair task performance but did not reduce cortical activity, challenging the idea that SC selects the target of visual attention.

Instead of searching for another correlate center of attention, Krauzlis and colleagues proposed a different model: *attention is an effect rather than a cause*. It would be the functional consequence of competition between “interpretations of the current state by the basal ganglia rather than competition to determine how sensory data is represented in the neocortex” (Krauzlis et al., 2014, p. 458). Such a reversal of how attention is intended realizes a “strange inversion of reasoning” (Dennett, 2009), where processes that do not have comprehension can nonetheless show competence. In Krauzlis case, attentive behaviors would not result from the comprehension of a homunculus selecting which sensory data is represented in neocortex. They would instead result from the competence of selective processes in the basal ganglia.

Further exploring this link prompts the identification of an interface—revolving around the concept of cell assembly (Hebb, 1949)—yielding a synergistic mapping between Dennett's Multiple-Drafts model (MDM) and Krauzlis' model regarding attention.

Dennett's MDM fundamental thesis is: “There is no single stream of consciousness, no place where ‘it all comes together' for a Central Meaner. There are multiple drafts, separate channels in which specialist circuits try to do their various things. Most of these fragmentary drafts of narrative play short-lived roles in the modulation of activity but some get promoted to further functional roles, in swift succession. The seriality of this process is not a hard-wired design feature, but rather the upshot of a succession of coalitions of these specialists” (Dennett, 1991, p. 253). These four sentences are the core building blocks of Dennett's explanation of consciousness. By analyzing each sentence, I will now build the interface and use it to map the two models (see Table 1 for a direct comparison).

**Table 1:** Interface providing a mapping between philosophical and neuroscientific terminology.

**Dennett terms**	**Interface**	**Krauzlis terms**
“Specialist”	Set of brain areas	
“Draft”	(specialist's) cell assembly	
“Affordance” (Gibson)	(object-action) cell assembly	“Candidate state”
“Coalition of specialists”	Dynamic mutual strengthening between (specialist's) cell assemblies, eventually leading to an (object-action) cell assembly	Strength of “different sensory inputs and types of knowledge contributions to the current state”
“Winning coalition”	Strengthened (object-action) cell assembly currently dominating the selection process	“Current state”
“Succession of coalitions”	Sequence of (object-action) cell assemblies (or phase sequence)	“Linked chain of states”

The first sentence rejects the homunculus, and Krauzlis shares it: “Viewing attention as an effect rather than a cause has the advantage of eliminating the need to find the homunculus that aims the spotlight of attention. Instead, we should be able to explain the functional circuits that give rise to the phenomenology of attention without using the word ‘attention”' (Krauzlis et al., 2014, p. 461).

In the second sentence, Dennett was intentionally vague using the term “draft.” A possible denotation for draft, however, may be the Hebbian cell assembly: “a diffuse structure comprising cells in the cortex and diencephalon (and also, perhaps, in the basal ganglia of the cerebrum), capable of acting briefly as a closed system, delivering facilitation to other such systems and usually having a specific motor facilitation” (Hebb, 1949, p. xix). As explicitly put in Hebb's definition, the concept of cell assembly identifies sets of cells, spanning the whole sensation-action arc. However, over time, two overlapping versions of the same concept emerged. *Object-action cell assemblies*, representing potential associations between sensory objects and actions (Humphreys and Riddoch, 2000), and (generic) *cell assemblies*, with a broader sense of “set[s] of neurons among which excitatory connections have been potentiated” (Harris, 2005). Therefore, if we denote Dennett's “specialist circuit” by a set of functionally related areas (e.g., visual: lateral geniculate nucleus, striate and extrastriate cortices, SC, …, as in Akins, 1996), then a “draft” can be denoted by an active (generic) cell assembly—one out of many that could be active at any given moment in the “specialist.”

The third sentence fits the proposed interface well, highlighting the fluidity of cell assemblies, and introducing a selection process involving them: “some get promoted to further functional roles, in swift succession.” But how, exactly?

This question is crucial for the MDM, and the interface may offer an answer, provided it succeeds in the terminology mapping. Krauzlis considers that “Good decision making depends crucially on properly identifying the current state of the animal and its environment” (Krauzlis et al., 2014, p. 457). This wording implies a “state” that has to be recognized. The concept of “state” has its root at the beginning of cybernetics, where every physical system (including an organism, its nervous system, and its environment) can be described by a finite set of states and state transitions (Ashby, 1961, p. 10). However, this powerful abstraction has been subsequently challenged because many relations between an organism and its environment, or “affordances” Gibson (2013), are equally possible in the same environmental state. The neural components of such relations have been mapped onto object-action cell assemblies (Humphreys and Riddoch, 2000; Cisek, 2007). Therefore, a distinction has to be made between Krauzlis' “candidate state”—denoted by any object-action cell assembly that can be potentially enacted—and his “current state” that should be denoted by an object-action cell assembly which has been selected, among many, to be actually enacted.

According to Krauzlis, the “current state” is evaluated by the basal ganglia. In fact, they receive sensory patterns (Voorn et al., 2004) and action plans (Jin et al., 2014) from cortex, threat detections from the amygdala (LeDoux, 2014), memories of action sequences from hippocampus (Jai and Frank, 2015) with their possible outcomes from prefrontal cortex (Donoso et al., 2014), and stimulus saliency from thalamic intralaminar nuclei (Ding et al., 2010; Doig et al., 2014). Each of these areas, in MDM terms, is a specialist producing a temporary draft. Within the basal ganglia, the striatum has the circuitry to implement competition among them (Tepper and Bolam, 2004; Cisek, 2007). However, “Because different sensory inputs and types of knowledge contribute unequally to different states, their influence on perception and action will be limited by the strength of their contribution to the state that currently dominates the competition” (Krauzlis et al., 2014, p. 458). This point is central to build the interface and realize the terminology mapping, thus it requires further unpacking.

The intrinsic recurrent connections between cells of an assembly stabilize their firing through mutual excitation (Harris, 2005) while also suppressing other assemblies through inhibition (Zenke et al., 2015). The occurrence of a specific assembly in one area both raises the probability of occurrence for some assemblies and quenches the probability of occurrence for some other assemblies in the same or other areas at some later time (Ponzi and Wickens, 2009; Truccolo et al., 2010) by means of short-term synaptic plasticity (von der Malsburg, 1994). Now, two or more specialists, each producing temporary drafts / cell assemblies, can interact through direct or striato-thalamo-cortical connections (Hikosaka et al., 2000; Sommer and Wurtz, 2004, 2006). These interactions, dynamically raising or lowering the probability of occurrence of an assembly given the occurrence of another, are the required mechanism for having dynamic coalitions of specialists' drafts, denoted by mutually strengthening assemblies. Eventually, one of these coalitions temporarily wins the competition in the striatum, and its entailed action is performed. The outcome for the organism is signaled, at the nervous system level, as dopaminergic reward (Threlfell et al., 2012) instrumental in selecting the synapses that will be targeted for growth (Li et al., 2003; Howe et al., 2013; Pelosi and Guarino, 2015), which will shape cell assemblies, and which will turn into (possibly better) behaviors (Montague et al., 1996).

With “state that currently dominates the competition” being the object-action cell assembly currently dominating the selection process in the basal ganglia (Dennett's winning coalition), we may now proceed to the fourth sentence, regarding the seriality and succession of coalitions. The proposed interface still matches: “As circumstances change, a candidate state that provides a poor match in one round of competition could emerge as predominant just moments later. This results in a linked chain of states, where the transition from one to the next is precipitated by some event or change in an internal variable, that gives the new state more support than the preceding one and carries along with a new decision policy” (Krauzlis et al., 2014, p. 458). Here, “candidate state” is denoted, as proposed earlier, by no more than an object-action cell assembly competing with all the others in the striatum. These candidate states, with their associated potential actions, represent different decision policies. Hence, a succession of selected states — comprehension-less but competence-full, thanks to a reinforcement learning process—steers the policy making that we label as “attentional” behavior. The reinforcement behind this succession of states, and its link to attentional behavior, is also fitting the Hebbian “phase sequence” (Hebb, 1949, p. 87).

To summarize (see Table 1), several cell assemblies (Dennett's “short-lived,” “coalitions of specialists,” “drafts,” or Krauzlis' “candidate states”) are dynamically strengthened and compete in the striatum. At every moment, one object-action cell assembly is temporarily dominating the selection process in the striatum (Dennett's winning “coalition,” or Krauzlis' “current state”), getting enacted. In time, a series of winning assemblies (Dennett's “succession of coalitions of specialists,” or Krauzlis' “linked chain of states”) gets selected to be pursued with actions, leading (or not) to rewards for the organism, ultimately promoting the formation, dissolution, and change of cell assemblies. Hence it is not a homunculus, rather learned associations that drive the production of “attentional” behaviors.”

The interface I proposed, here, and the mapping between the terminology used by Dennett and that of Krauzlis is meant to encourage the exchange between philosophy of mind and neuroscience. In fact, on the one hand, it seems that the philosophical refinement of questions regarding the mind needs more scientific injections (Dennett, 2003). While, on the other hand, neuroscience is in danger of a technological stale-point because it is lacking more encompassing and purposeful views (Frégnac, 2017). If, and only if, a common terminology will be adopted such an exchange will take place and benefit both disciplines.

## Author contributions

The author confirms being the sole contributor of this work and approved it for publication.

### Conflict of interest statement

The author declares that the research was conducted in the absence of any commercial or financial relationships that could be construed as a potential conflict of interest.
